# Supplementary figures and images for: A preliminary study of the salivary microbiota of young male subjects before, during, and after acute high-altitude exposure
Source: PeerJ. 2023 Jun 27;11:e15537. doi: 10.7717/peerj.15537 (PMC10312199; doi:10.7717/peerj.15537)

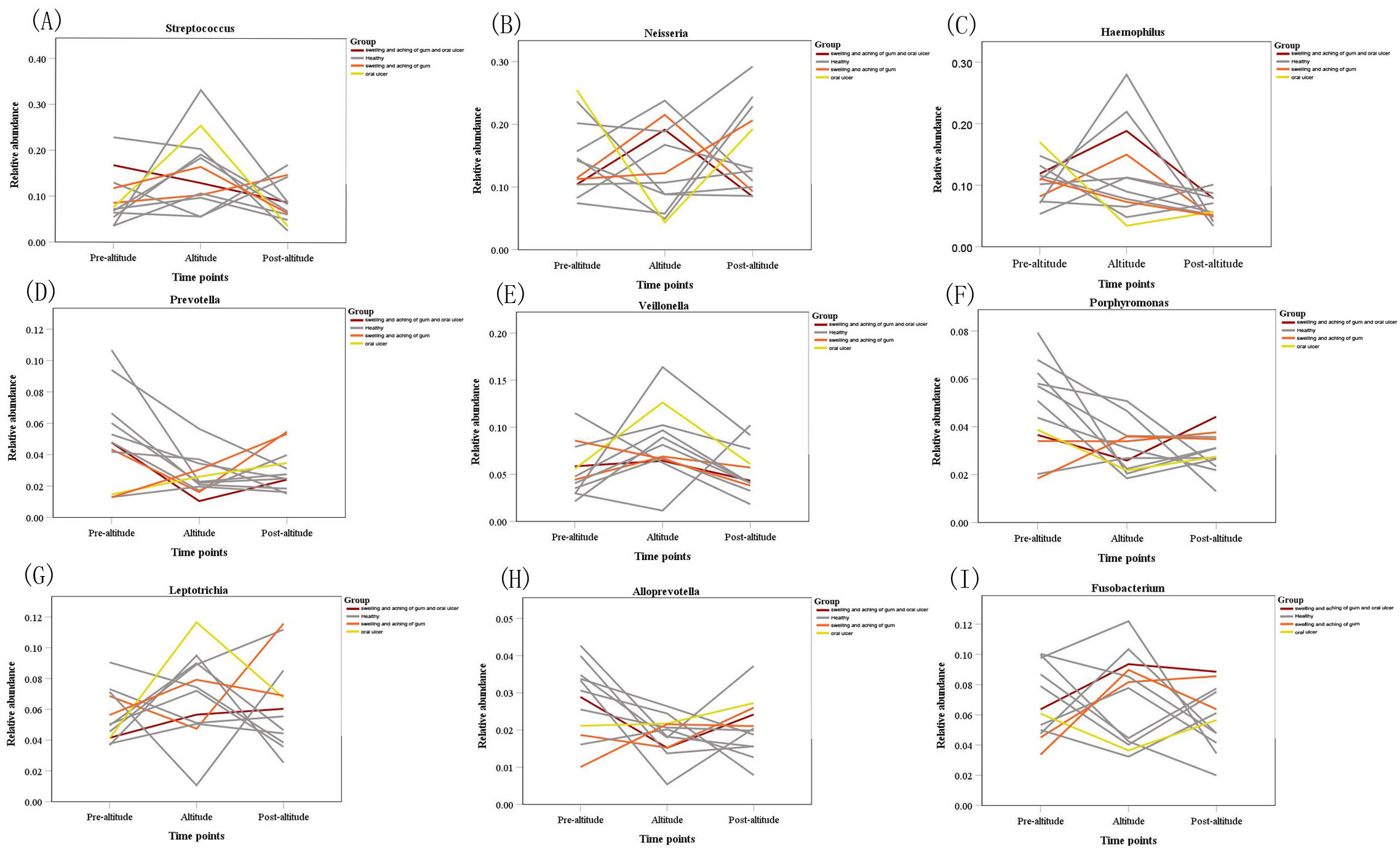

Supplement: Supplemental Information 1 — The colours in each figure represent different subjects experiencing different symptoms, as indicated in the figure legend. The red color indicates that the subject had swollen gums and oral ulcer after entering the plateau. The gray colour indicates that subjects were healthy after entering the plateau. The orange color indicates that the subject had swollen gums after entering the plateau. The yellow color indicates that the subject had oral ulcer after entering the plateau. [file peerj-11-15537-s001.png]

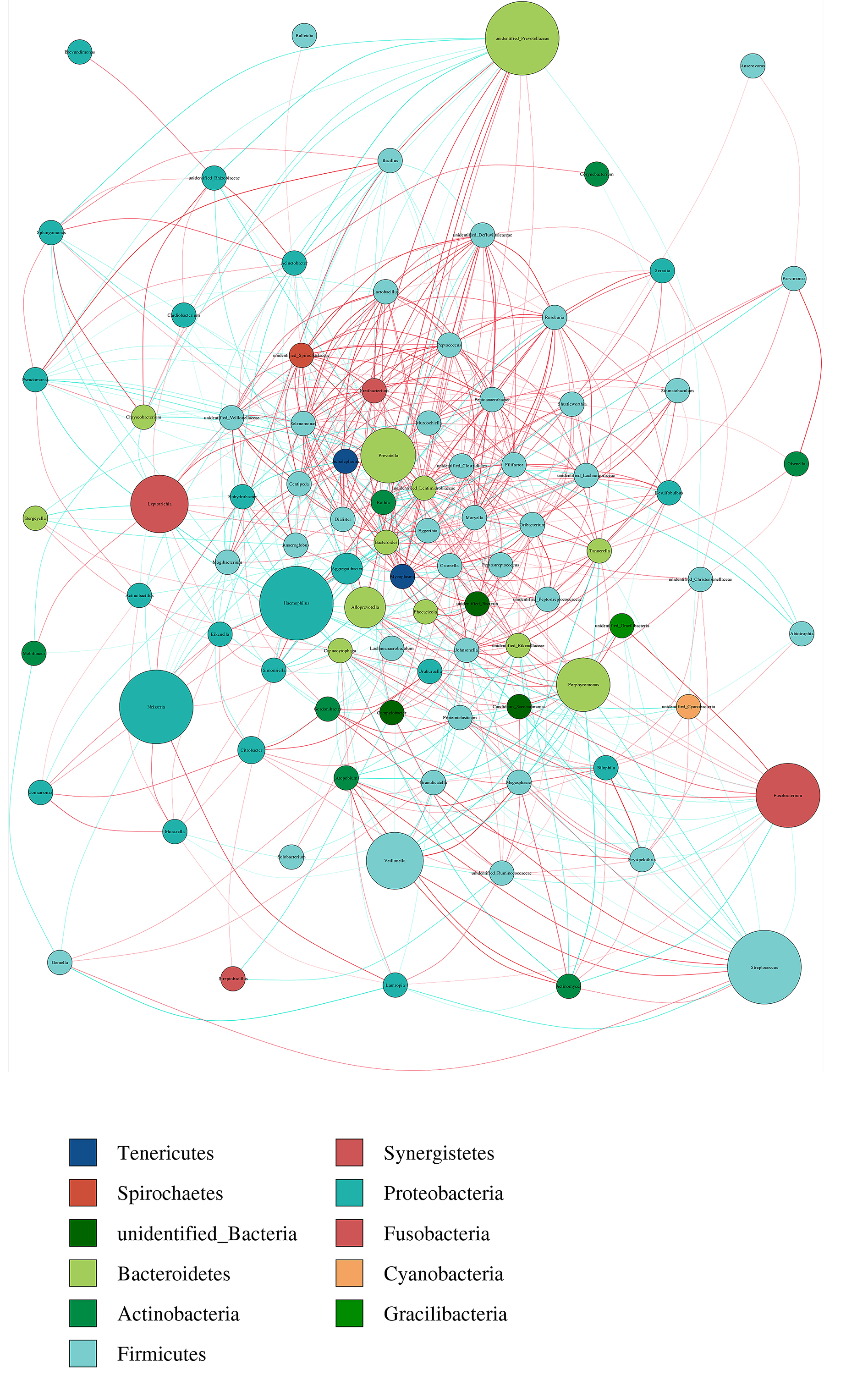

Supplement: Supplemental Information 2 — A connection shows a strong and significant correlation (∥R ∥ > 0.6). The width of the line between nodes is positively correlated with the absolute value of the correlation coefficient of the species interaction. The red and blue lines indicate positive correlations and negative correlations, respectively. [file peerj-11-15537-s002.png]

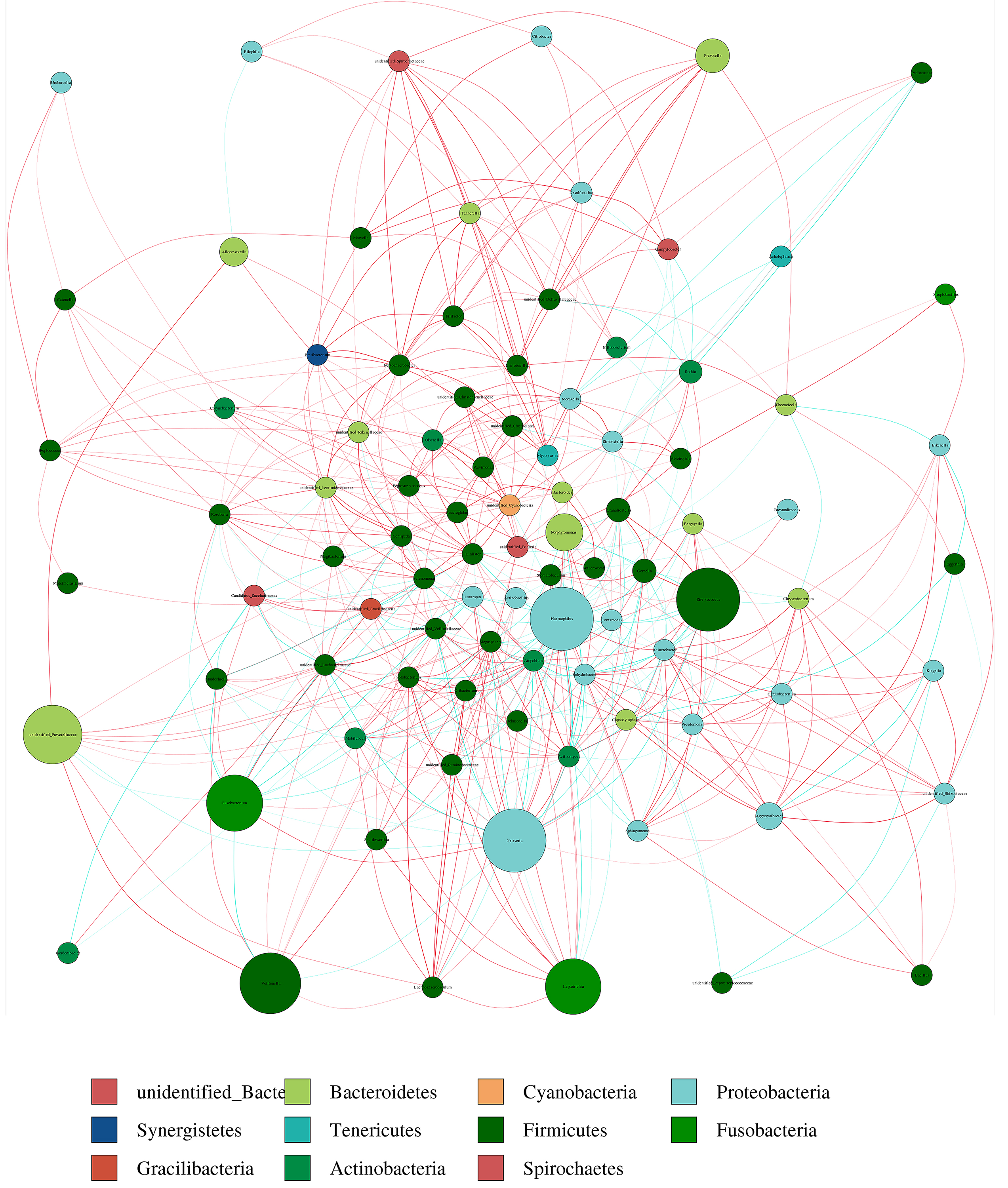

Supplement: Supplemental Information 3 — A connection shows a strong and significant correlation(∥R ∥ > 0.6). The width of the line between nodes is positively correlated with the absolute value of the correlation coefficient of the species interaction. The red and blue lines indicate positive correlations and negative correlations, respectively. [file peerj-11-15537-s003.png]
