# Supplementary material for: A preliminary study of the salivary microbiota of young male subjects before, during, and after acute high-altitude exposure
Source: PeerJ. 2023 Jun 27;11:e15537. doi: 10.7717/peerj.15537 (PMC10312199; doi:10.7717/peerj.15537)
Supplement: Supplemental Information 4 — Y: Positive symptoms; N: Negative symptoms; Pre-: pre-altitude; In-: altitude; Post-: post- altitude [file peerj-11-15537-s004.docx]

**Table S1** Oral health status survey of 12 subjects during acute high-altitude exposure

| Category  Number | Age | Bleeding when brushing | | | Gum swelling and aching | | | Tooth pain | | | Temporomandibular joint discomfort when eating | | | Oral ulcer | | | | Other mucosal discomfort | | |
| --- | --- | --- | --- | --- | --- | --- | --- | --- | --- | --- | --- | --- | --- | --- | --- | --- | --- | --- | --- | --- |
|  |  | Pre- | In- | Post- | Pre- | In- | Post- | Pre- | In- | Post- | Pre- | In- | Pos- | Pre- | In- | Pos- | Pre- | | In- | Post- |
| 1 | 29 | N | N | N | N | Y | N | N | N | N | N | N | N | N | Y | N | N | | N | N |
| 2 | 28 | N | N | N | N | N | N | N | N | N | N | N | N | N | N | N | N | | N | N |
| 3 | 30 | N | N | N | N | Y | N | N | N | N | N | N | N | N | N | N | N | | N | N |
| 4 | 27 | N | N | N | N | N | N | N | N | N | N | N | N | N | N | N | N | | N | N |
| 5 | 44 | N | N | N | N | N | N | N | N | N | N | N | N | N | N | N | N | | N | N |
| 6 | 26 | N | N | N | N | N | N | N | N | N | N | N | N | N | N | N | N | | N | N |
| 7 | 45 | N | N | N | N | N | N | N | N | N | N | N | N | N | N | N | N | | N | N |
| 8 | 31 | N | N | N | N | N | N | N | N | N | N | N | N | N | N | N | N | | N | N |
| 9 | 39 | N | N | N | N | N | N | N | N | N | N | N | N | N | N | N | N | | N | N |
| 10 | 35 | N | N | N | N | N | N | N | N | N | N | N | N | N | N | N | N | | N | N |
| 11 | 42 | N | N | N | N | N | N | N | N | N | N | N | N | N | Y | N | N | | N | N |
| 12 | 43 | N | N | N | N | Y | N | N | N | N | N | N | N | N | N | N | N | | N | N |

Y: Positive symptoms; N: Negative symptoms; Pre-: pre-altitude; In-: altitude; Post-: post-altitude
