# Supplementary material for: A preliminary study of the salivary microbiota of young male subjects before, during, and after acute high-altitude exposure
Source: PeerJ. 2023 Jun 27;11:e15537. doi: 10.7717/peerj.15537 (PMC10312199; doi:10.7717/peerj.15537)
Supplement: Supplemental Information 5 — (1) Sample names refer to the 36 samples, (A): pre-altitude samples; (B): altitude samples; (C): post-altitude samples. (2) Raw PE is the first data read out by the sequencing platform; (3) Raw tags refers to the sequence after splicing primer fragments from the original data; (4) Clean tags indicate a high-quality sequence with an appropriate length obtained by filtering raw tags; (5) Effective tags refers to the sequences in clean tags that do not have chimeras and can be used for in-depth analysis; (6) Base is the statistics of nucleotide bases in valid data; (7) AVG len refers to the average length of valid data; (8–9) Q20 and Q30 refer to the base ratio with base quality values greater than 20 (sequencing error rate < 1%) and 30 (sequencing error rate < 0.1%) in the valid data, respectively; (10) GC % represents the content of GC bases in the effective sequence; (11) Effective % refers to the ratio of effective data to the original number of offline users %. [file peerj-11-15537-s005.docx]

**Table S2** Data preprocessing statistics and quality control results of 36 saliva samples

| Sample Name | Raw PE | Raw Tags | Clean Tags | Effective Tags | Base | Avg  len | Q20 | Q30 | GC% | Effective% |
| --- | --- | --- | --- | --- | --- | --- | --- | --- | --- | --- |
| A1 | 99,299 | 92,967 | 88,130 | 66,587 | 28,178,479 | 423 | 97.74 | 93.25 | 51.69 | 67.06 |
| A2 | 93,132 | 88,612 | 83,958 | 64,093 | 27,121,377 | 423 | 98.05 | 93.97 | 51.67 | 68.82 |
| A3 | 94,539 | 89,234 | 84,441 | 62,570 | 26,442,050 | 423 | 97.90 | 93.64 | 52.57 | 66.18 |
| A4 | 89,536 | 83,097 | 79,250 | 60,214 | 25,392,073 | 422 | 97.97 | 93.86 | 51.62 | 67.25 |
| A5 | 97,564 | 91,982 | 88,439 | 62,687 | 26,467,283 | 422 | 98.07 | 93.98 | 51.57 | 64.25 |
| A6 | 83,132 | 78,559 | 75,019 | 60,567 | 25,451,490 | 420 | 97.96 | 93.73 | 51.45 | 72.86 |
| A7 | 90,472 | 84,999 | 80,763 | 66,471 | 27,986,364 | 421 | 97.83 | 93.44 | 51.44 | 73.47 |
| A8 | 80,344 | 76,085 | 73,035 | 64,386 | 27,127,437 | 421 | 98.02 | 93.92 | 51.48 | 80.14 |
| A9 | 85,170 | 80,301 | 76,379 | 64,643 | 27,296,462 | 422 | 98.18 | 94.24 | 51.61 | 75.90 |
| A10 | 88,325 | 82,331 | 78,462 | 69,281 | 29,418,493 | 425 | 97.75 | 93.31 | 52.07 | 78.44 |
| A11 | 91,418 | 87,212 | 82,975 | 67,601 | 28,695,504 | 424 | 98.07 | 94.00 | 52.05 | 73.95 |
| A12 | 83,420 | 78,982 | 74,502 | 65,411 | 27,740,644 | 424 | 97.95 | 93.75 | 52.96 | 78.41 |
| B1 | 84,114 | 79,022 | 75,621 | 64,697 | 27,398,907 | 423 | 97.95 | 93.71 | 51.85 | 76.92 |
| B2 | 96,374 | 89,184 | 85,111 | 65,827 | 27,855,794 | 423 | 97.96 | 93.77 | 52.03 | 68.30 |
| B3 | 82,156 | 78,055 | 75,358 | 62,286 | 26,389,247 | 424 | 98.07 | 93.96 | 51.95 | 75.81 |
| B4 | 96,558 | 91,849 | 88,142 | 61,354 | 25,912,515 | 422 | 97.91 | 93.64 | 51.85 | 63.54 |
| B5 | 99,123 | 93,407 | 88,993 | 61,690 | 26,066,591 | 423 | 98.04 | 93.96 | 51.89 | 62.24 |
| B6 | 72,864 | 68,780 | 65,323 | 58,468 | 24,772,800 | 424 | 98.15 | 94.18 | 52.00 | 80.24 |
| B7 | 84,702 | 79,648 | 75,896 | 64,860 | 27,483,896 | 424 | 97.81 | 93.54 | 51.89 | 76.57 |
| B8 | 73,864 | 69,878 | 67,798 | 64,474 | 27,305,797 | 424 | 98.10 | 94.02 | 51.78 | 87.29 |
| B9 | 74,970 | 71,314 | 67,396 | 59,467 | 25,107,949 | 422 | 97.96 | 93.85 | 52.72 | 79.32 |
| B10 | 86,026 | 80,276 | 75,557 | 67,236 | 28,333,945 | 421 | 97.87 | 93.61 | 51.56 | 78.16 |
| B11 | 94,898 | 85,438 | 79,979 | 69,209 | 29,197,300 | 422 | 97.77 | 93.42 | 51.84 | 72.93 |
| B12 | 83,715 | 78,782 | 74,676 | 65,795 | 27,741,901 | 422 | 97.91 | 93.68 | 51.65 | 78.59 |
| C1 | 85,139 | 80,037 | 75,751 | 65,758 | 27,557,304 | 419 | 97.91 | 93.70 | 51.63 | 77.24 |
| C2 | 93,496 | 88,898 | 84,714 | 67,765 | 28,485,786 | 420 | 98.01 | 93.94 | 51.61 | 72.48 |
| C3 | 94,759 | 89,461 | 84,277 | 60,625 | 25,487,697 | 420 | 97.89 | 93.68 | 51.60 | 63.98 |
| C4 | 80,433 | 75,393 | 72,111 | 62,793 | 26,569,700 | 423 | 97.90 | 93.71 | 52.28 | 78.07 |
| C5 | 83,608 | 79,511 | 75,472 | 66,856 | 28,322,503 | 424 | 98.05 | 93.96 | 52.38 | 79.96 |
| C6 | 81,067 | 76,222 | 72,591 | 63,123 | 26,723,675 | 423 | 97.90 | 93.64 | 53.82 | 77.87 |
| C7 | 86,501 | 80,181 | 75,927 | 66,927 | 28,264,997 | 422 | 97.91 | 93.68 | 52.18 | 77.37 |
| C8 | 91,025 | 78,472 | 73,705 | 63,374 | 26,696,854 | 421 | 97.93 | 93.76 | 52.18 | 69.62 |
| C9 | 83,373 | 78,665 | 75,726 | 61,160 | 25,847,312 | 423 | 98.11 | 94.09 | 52.29 | 73.36 |
| C10 | 83,963 | 78,987 | 75,446 | 60,173 | 25,150,142 | 418 | 97.99 | 93.84 | 52.35 | 71.67 |
| C11 | 83,147 | 78,735 | 75,356 | 60,360 | 25,436,895 | 421 | 97.95 | 93.80 | 52.19 | 72.59 |
| C12 | 85,825 | 80,270 | 76,542 | 60,201 | 25,371,113 | 421 | 98.00 | 93.90 | 52.20 | 70.14 |

(1) Sample names refer to the 36 samples, A: pre-altitude samples; B: altitude samples; C: post-altitude samples. (2) Raw PE is the first data read out by the sequencing platform; (3) Raw tags refers to the sequence after splicing primer fragments from the original data; (4) Clean tags indicate a high-quality sequence with an appropriate length obtained by filtering raw tags; (5) Effective tags refers to the sequences in clean tags that do not have chimeras and can be used for in-depth analysis; (6) Base is the statistics of nucleotide bases in valid data; (7) AVG len refers to the average length of valid data; (8-9) Q20 and Q30 refer to the base ratio with base quality values greater than 20 (sequencing error rate < 1%) and 30 (sequencing error rate < 0.1%) in the valid data, respectively; (10) GC % represents the content of GC bases in the effective sequence; (11) Effective % refers to the ratio of effective data to the original number of offline users %.
