# Supplementary material for: A preliminary study of the salivary microbiota of young male subjects before, during, and after acute high-altitude exposure
Source: PeerJ. 2023 Jun 27;11:e15537. doi: 10.7717/peerj.15537 (PMC10312199; doi:10.7717/peerj.15537)
Supplement: Supplemental Information 6 — ND (network diameter): the maximum measurement length of the diameter of the network diagram.There is the shortest distance between any two points, and the extent of these distances is the diameter of the network diagram. MD (modularity degree): network diagram modularity degree measures the modularity degree of the network group structure and describes the rationality of the network being divided into different modules or the differentiation between different modules. CC (clustering coefficient): the possibility that the adjacent nodes of the node are connected with each other, and the connectivity of the network graph is the average of the connectivity of all nodes. GD (graph density): network graph density is the actual number of edges divided by the number of all possible edges. AD (average degree): average connectivity is the number of edges connected by the node. The average connectivity is the sum of the connectivity of all nodes divided by the total number of nodes. APL (average path length): the mean distance. There is the shortest path between every two pairs of nodes. The average path length is the sum of these distances of all pairs of nodes divided by the number of node pairs. [file peerj-11-15537-s006.docx]

**Table S3** Network parameters for the pre-altitude and altitude groups.

|  | ND | MD | CC | GD | AD | APL |
| --- | --- | --- | --- | --- | --- | --- |
| Pre-altitude | 9 | 0.377 | 0.485 | 0.074 | 14.909 | 3.363 |
| Altitude | 10 | 0.453 | 0.473 | 0.061 | 12.141 | 3.557 |

ND (network diameter): the maximum measurement length of the diameter of the network diagram. There is the shortest distance between any two points, and the extent of these distances is the diameter of the network diagram. MD (modularity degree): network diagram modularity degree measures the modularity degree of the network group structure and describes the rationality of the network being divided into different modules or the differentiation between different modules. CC (clustering coefficient): the possibility that the adjacent nodes of the node are connected with each other, and the connectivity of the network graph is the average of the connectivity of all nodes. GD (graph density): network graph density is the actual number of edges divided by the number of all possible edges. AD (average degree): average connectivity is the number of edges connected by the node. The average connectivity is the sum of the connectivity of all nodes divided by the total number of nodes. APL (average path length): the mean distance. There is the shortest path between every two pairs of nodes. The average path length is the sum of these distances of all pairs of nodes divided by the number of node pairs.
